# Universal protocol for the wafer-scale manufacturing of 2D carbon-based transducer layers for versatile biosensor applications

**DOI:** 10.1016/j.mex.2023.102402

**Published:** 2023-09-27

**Authors:** Xiaoling Lu, Walid-Madhat Munief, Pavel Damborský, Alice Kasjanow, Jaroslav Katrlík, Vivek Pachauri, Sven Ingebrandt

**Affiliations:** a Department of Informatics and Microsystem Technology, University of Applied Sciences Kaiserslautern, Amerikastrasse 1, Zweibruecken 66482, Germany; b Institute of Chemistry, Slovak Academy of Sciences, Dúbravská cesta 9, Bratislava, Slovakia; c RAM DE GmbH. Science Park 2, Saarbruecken 66123, Germany; d Institute of Materials in Electrical Engineering 1, RWTH Aachen University, Sommerfeldstrasse 24, Aachen 52074, Germany; e Department of Physical Chemistry, Saarland University, 66123 Saarbruecken, Germany; f Micronit GmbH, Konrad-Adenauer-Allee 11, 44263 Dortmund, Germany

**Keywords:** Graphene-oxide, Thin film, Gas-phase silanization, Lithography, Micro and nano-fabrication, Thermal reduction, Sensor, Wafer-scale processing of (reduced) GO thin-films (WSPgo)

## Abstract

In this manuscript, we present a comprehensive fabrication protocol for high-performance graphene oxide (GO) sensor concepts. It is suitable for a variety of biosensing applications and contains the essential process steps, starting with vapor phase evaporation for siloxane monolayers, followed by spin-coating of GO as a nanometer-thin transducer with exceptional homogeneity and micromechanical surface methods which enable seamless transformation of GO transducers to be desired micro and nano dimensions.

In addition to linking basic research and innovative sensor concepts with an outlook for commercial applications of point-of-care systems for early-stage diagnostics, the authors consider it necessary to take a closer look at the manufacturing processes to create more transparency and clarity, to manufacture such specific sensor concepts systematically. The detailed manufacturing approaches are intended to motivate practitioner to explore and improve this GO-based key technology.

This process development is illustrated below using the manufacturing methods for three types of sensors, namely sensors based on i) surface plasmon resonance spectroscopy (SPR), ii) impedance spectroscopy and iii) bio-field effect transistors (ISFETs).

The obtained results in this work prove successful GO sensor productions by achieving:

• Uniform and stable immobilization of GO thin films,
• High yield of sensor units on a wafer scale, here up to 96 %,
• Promising integration potential for various biomedical sensor concepts to early-stage diagnostic.

## Graphical abstract

**Figure:**
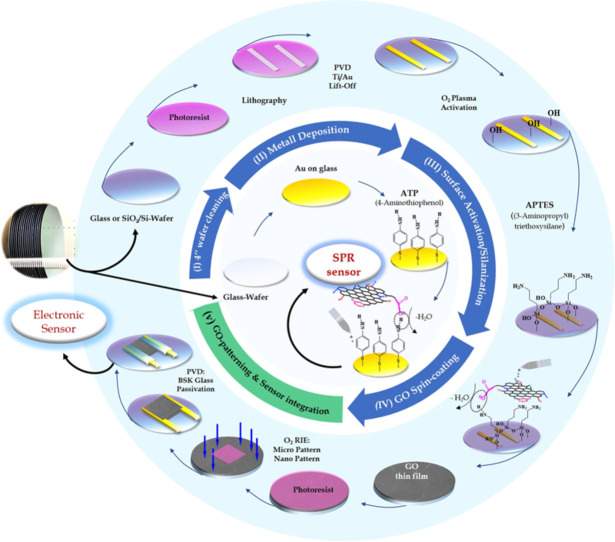

Specifications table

**Table:** 

Subject area:	Materials Science
More specific subject area:	2D materials for sensor applications
Name of your method:	Wafer-scale processing of (reduced) GO thin-films (WSPgo)
Name and reference of original method:	N/A
Resource availability:	N/A

## Method details

The development of technological novel sensor systems for point-of-care testing is tightly paired with constantly increasing competition market positions, and cost efficiency with a high-yield production. It is also often the case that not every technical innovation from the development stage, no matter how brilliant, leads to a market entry. All scientists face such challenges in research and development departments worldwide. In this respect, new interdisciplinary sensor developments are addressed, involving know-how and multi scientific disciplines such as organic/inorganic chemistry, biomolecular chemistry, electrochemistry, surface/interface chemistry, micro/nanosystems technology, optics/electronics and microfluidics. Our scientific work presents all production steps to illustrate that the cost-effective manufacture of such a sensor concept is truly feasible with existing technologies. Therefore, we made it our task to give priority to pure sensor fabrication in this manuscript and presented a comprehensive fabrication protocol that mainly deals with the wafer-scale fabrication of (r)GO transducer [1,2] for biosensor applications exemplified in SPR [3], impedance spectroscopy [4,5] and ion-sensitive field-effect transistors (ISFETs) [6,7] configurations. These established works serve as a comprehensive universal fabrication guideline for readers. The quality and functionality of the rGO based biosensors have been tested and evaluated by detecting protein (ex. lectin ConA), antigen (ex. prostate specific antigen (PSA)), glucose, peptide (ex. cardio markers such as N-terminal pro-B-type natriuretic peptide (NT-Pro-BNP)) and DNA (ex. single nucleotide polymorphisms). The aim of this work is to give the readers a technological overview about fabrication strategies of how a powerful, micro/nano scalable and highly sensitive GO transducer can be developed from the disperse GO solution to novel types of sensors, which new avenues in the field for biomedical research studies are enabled.

**Fig. 1:**
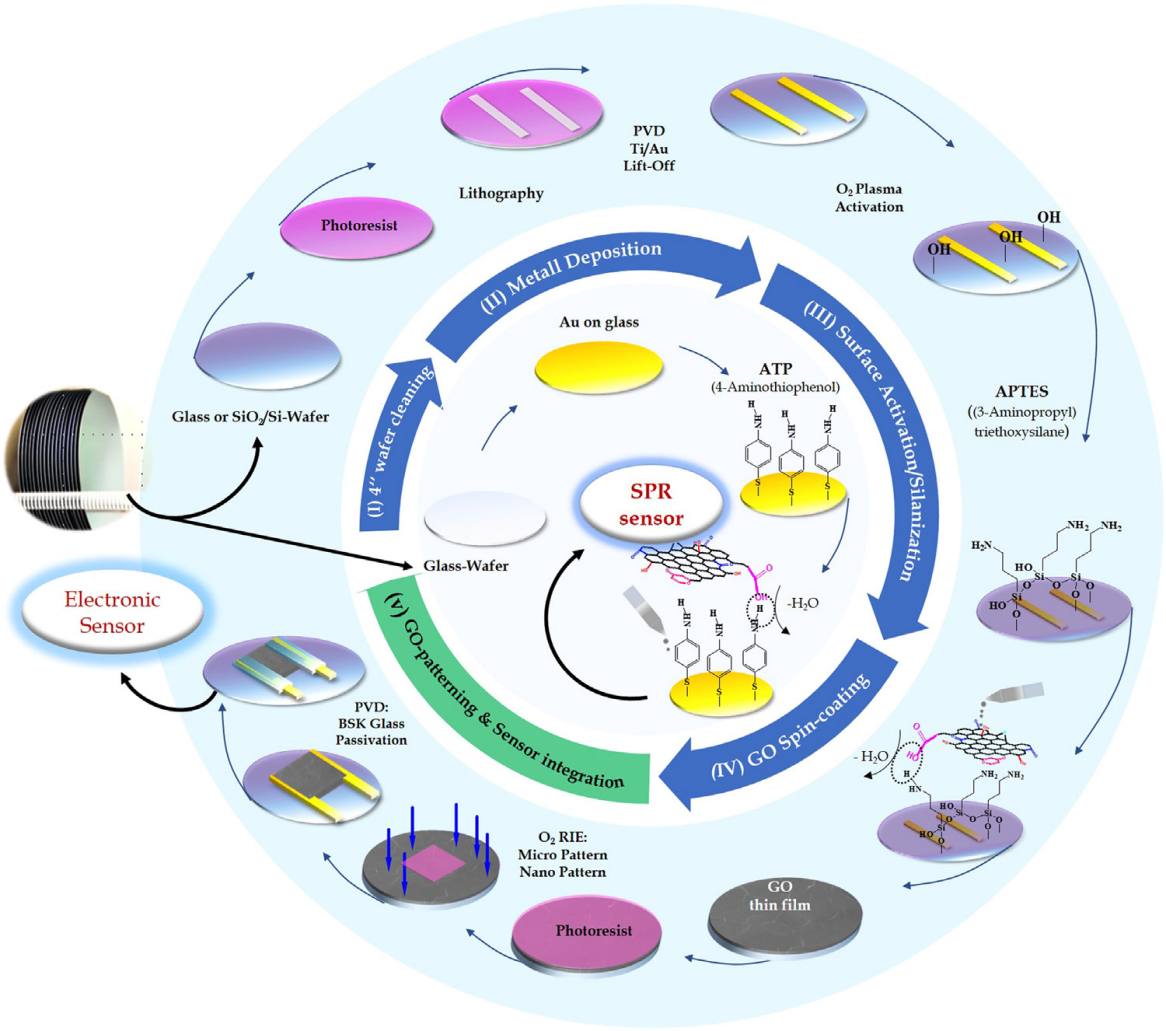
Scalable preparation flow for the GO thin film across diverse substrates, using a GO stock aqueous solution. After the appropriate surface treatment (step I, II) of the substrates and immobilization of linker molecules (step III), the GO nanolayer is realized. On one side, the functional groups of the linker molecules form robust bonds with the substrate, leaving amino groups exposed to the environment. For the gold wafer, the linker molecule aminothiophenol (ATP) was utilized, with thiol groups forming strong bonds to the gold substrate. In both instances, the free amino groups react with the carboxyl (-COOH) groups on the GO flakes, forming a strong binding force and establishing a thin film (step IV). For a glass or silicon substrate, a linker molecule such as aminopropyltriethoxysilane (APTES) can robustly bind to the hydroxyl (-OH) groups on the substrate following an oxygen (O_2_) plasma treatment. The micro/nano-structuring technique is deployed to create microelectrodes, GO patterns, and a passivation layer on the microelectrodes (step V).

The protocol includes pre-processing of the rigid and flexible substrates [8] by physical and/or chemical cleaning/activation and gas-phase silanization with aminothiophenol (ATP) or (3-aminopropyl)triethoxsilane (APTES) [1,[9], [10], [11], [12]]. By spin-coating GO aqueous solution onto the condensed ATP modified Au substrate or aminopropylsiloxane (APS) modified wafers, the functional groups of GO, specifically the tertiary alcohols and ethers, form a covalent bond between the GO flakes and the amino groups of modified 4-inch substrates, resulting in GO transducers layer thicknesses between 1 and 3 nm. Thermal treatment steps hereafter transform the insulator state of the GO transducer into reduced rGO semi-metallic thin films. So far, the former Au/ATP/ GO chips are ready for electrical SPR biosensor application [3]. The latter substrate/APS/ GO chips still require extra process steps before utilization: based on the predefined structures on photomask, surface micromachining and lithography techniques enable micro and nano scale patterning. These successfully integrated rGO thin films are electrically contacted with Ti/Au electrodes. The conductor tracks are hermetically sealed with a highly stable borosilicate glass (BSK) passivation so that this production is perfect for sensor applications in high ionic-strength solutions in which electrical impedance spectroscopy [4,5] and ISFETs [6,7] can operate. Fig. 1 presents a schematic illustration delineating both process flows, thereby highlighting the distinct differences between these two types of device platforms.

1. In the following chapters, the process lines, equipment, reagents, and methods for the wafer-scale fabrication of thin GO films on different substrates, as well as their micro- and nano structuring, including metallization and BSK glass passivation, are systematically explained in chronological order. Major equipment used, reagents for gas phase silanization and reagents for GO thin film preparation are listed in Tables 1, 2 and 3, respectively.

**Table 1:** Major equipment.

Equipment	Model	Company	Country
Spin coater/Hot plate	Delta 30 T2	Süss	Germany
Mask aligner	MA/BA 6	Süss	Germany
Reactive ion etching (RIE)	SI 591 M	SENTECH Instruments GmbH	Germany
Plasma stripper	300-E	Technics Plasma	USA
Electron Beam Evaporator	BAK500	Oerlikon Balzers	Liechtenstein
High temperature oven	DS-3900-PC-150	INOTHERM	Germany
Vacuum oven	VT5042 EKP	Heraeus	Germany
Wafer dicing saw	DAD-2H/6T	Disco	Japan
Glovebox	GS GLOVEBOX	Systemtechnik GmbH	Germany

**Table 2:** Reagents for gas phase silanization.

Name of chemical	Company	Country	CAS Number
(3-Aminopropyl) triethoxysilane (APTES)	Sigma Aldrich	Germany	919–30–2
4-aminothiopheol (4-ATP)	Sigma Aldrich	Germany	123–30–8

**Table 3:** Reagents for GO thin film preparation.

Name of chemical	Company	Country	CAS Number
GO aqueous solution	self-made in the lab [2]	–	–
Acetone	Sigma Aldrich	Germany	67–64–1
Isopropanol	Sigma Aldrich	Germany	67–63–0

## Substrate cleaning and activation

The establishment of seamless 2D thin films necessitates cleaning, and partially activating, surfaces of gold, silicon, or glass wafers. Activation heightens the density of hydroxyl groups—an essential step for optimal gas-phase silanization, particularly when aiming to form a continuous layer over expansive areas. For silicon wafers, a dry oxide insulator of 140 nm thickness is recommended to circumvent leakage currents between the silicon substrate and the electrode system used. Two methods—physical and chemical—have been established for the cleaning and activation of pure silicon, SiO_2_/Si, or glass wafers. Both methods of surface activation of the solid work very well and can be chosen depending on the equipment available.

### Physical method for surface treatment

The plasma process in an oxygen atmosphere provides an effective and reproducible means for cleaning and activating wafer surfaces without requiring rinse steps with aqueous solutions. For gold surfaces, this process primarily aims to remove physically adsorbed contaminants. For glass or silicon substrates, it enhances the transient formation of hydroxyl groups, serving as surface anchors for the silanes, thereby facilitating optimal monolayer formation.

This plasma process occurs under vacuum conditions. Oxygen radicals, that form during activation, briefly exist on the wafer surfaces. These radicals can undesirably trigger side reactions with the silanes' inorganic side chains, potentially compromising the quality of the layer formation. Following this treatment, it is recommended to wait for 10 min before initiating gas-phase silanization. Process parameters of physical treatment are shown in Table 4.

**Table 4:** Process parameters of physical treatment.

Process	Material	Parameters
Plasma stripper	O_2_	40 % O_2_, 50 W, 30 s (PVA TePla AG - Germany)

NOTE: When activation and silanization are performed in a reactor chamber (i.e., under continuous vacuum without chamber venting) a waiting period of 30 min is advised. This duration corresponds to the lifetime of oxygen radicals under vacuum conditions on the reactor walls. If the silane is introduced prematurely, the oxygen radicals can provoke undesired side reactions that can adversely impact the layer quality of the siloxane monolayers on the solid surfaces.

### Chemical method for surface treatment

The second approach involves wet-chemical cleaning and activation. This technique, compared to plasma treatment, necessitates greater expertise and practical experience from the user. Regarding industrial scalability, the focus should be on standardization, accurate documentation, and process management. Silicon wafers are immersed in a bath of Caro's acid, with the aim of eliminating both organic and inorganic contaminants while simultaneously activating the surface. Subsequently, the wafers must be thoroughly rinsed with deionized (DI) water and dried under a nitrogen stream for 1 minute. Process parameters of chemical treatment are shown in Table 5.

**Table 5:** Process parameters of chemical treatment.

Process	Material	Parameters	CAS Number
Spin Rinse Dryer (SRD)	DI-water N_2_-flow	1700 rpm for 3 min 2000 rpm for 4 min	
Caro acid	H_2_SO_4_ 98 wt%; H_2_O_2_ 30 wt% [mixing ratio 1:3]	*T* = 120 °C, *t* = 20 min	7722–84–1 7664–93–9
Quick Dump Rinse (QDR)	DI-water N_2_-flow	1700 rpm for 3 min 2000 rpm for 4 min	

NOTE: The physical and chemical treatments can be applied to glass wafers, silicon wafers with or without dry oxide and Silicon-on-Insulator (SOI).

### Optional dry oxide on Si wafer

The silicon wafers can be directly transferred to a high-temperature oxidation furnace and thermally oxidized in an O_2_ atmosphere. Following the production of the dry oxide, the physical or chemical methods can be utilized for the upcoming silanization steps. Process parameters of thermal dry oxidation are shown in Table 6.

**Table 6:** Process parameter of thermal dry oxidation.

Process	Material	Parameters
Oxidation furnace	O_2_	*T* = 1050 °C, *t* = 3.5 h, O_2_ flow = 2 sccm (Inotherm diffusion furnace DS-3 900 PC/150-Germany)

### Waferscale fabrication of Au thin-film, microelectrodes and passivation layer

#### Lithography steps (for electronic device, mask type 1)

The subsequent lithographic process for fabricating conductive tracks consists of photoresist spin-coating, exposure, development, as well as the process steps of vapor deposition and photoresist stripping (Table 7).

**Table 7:** Process steps and parameters for wafer manufacturing (transferring the structures of mask type 1).

Process	Material	Parameters
Spin Coater	Image reversal photoresist AR-U 4030	3 ml 7 s, 100 rpm 60 s, 3000 rpm (Thickness: 1.7 µm)
Hotplate		85 °C; 2 min Rest for 10 min
Mask aligner	Mask type 1	10 s, 6 mW, hard contact mode
Hotplate		115 °C, 5 min Rest for 10 min
Mask aligner	Flood exposure	25 s, 6 mW
Reverse development	AR 300–26; DI	1:3, ca. 10 s
SRD	DI N_2_	700 rpm, 3 min 2000 rpm, 4 min
Microscope and FTP		Structure control

NOTE: Visual inspection for the photoresist structure with a microscope and Film Thickness Probe (FTP) is recommended.

#### Physical Vapor Deposition (PVD) metal coating (for SPR and electronic devices)

High-purity metals, namely titanium (or chromium) and gold, were deposited onto bare glass substrate or the developed photoresist using the Physical Vapor Deposition (PVD) technique for either SPR or electronic devices, following the process parameters listed in Table 8.

**Table 8:** Process steps and PVD-parameters.

Process	Material	Parameters
Plasma stripper	O_2_	40 % O_2_, 50 W, 0,5 min
PVD metallization	Ti, Au	a) Optical SPR units: Ti: 3 nm, 0.2 nm/s, power 19 %; Au: 47 nm, 0.5 nm/s, power 24.5 % b) Electronic IDE units: Ti: 25 nm, 0.2 nm/s, power 19 %; Au: 250 nm, 0.5 nm/s, power 24.5 %

#### Lift off (for electronic device)

After the metal coating by PVD technique, the sacrificial stencil layer created by photoresist patterns is cleaned away together with metal layer upon by the following process listed in Table 9, while the metal layer contacting directly with the substrate remains.

**Table 9:** Process parameters for the lift off.

Process	Material	Parameter
Acetone cleaning	Acetone Beaker 1 Acetone Beaker 2	5 min; 60 W 1 min; fresh Acetone
Cleaning bench	Isopropanol	Rinse off
QDR	DI-water	2 min
SRD	DI N_2_	700 rpm; 3 min 2000 rpm; 4 min
Plasma stripper	O_2_	40 % O_2_; 350 W; 5 min

#### Lithography and PVD for metallic conductive line passivation of borosilicate glass (for electronic device, mask type 2)

The metallic conductor tracks are passivated with the mask type 2. Only the interdigitated electrode (IDE) connections are not passivated so that they are later in direct contact with the aqueous analytes. With the passivation mask, 92 % of the conductor track area is electrically and hermetically insulated by a BSK glass layer. The advantage of this type of passivation is the reduction of leakage currents during the performance of the bioassay. Respective lithography steps are listed in Table 10.

**Table 10:** Process steps and parameters for wafer manufacturing (transferring the structures of mask type 2).

Process	Material	Parameter
Spin coater	Image reversal photoresist AR-U 4030	3 ml 7 s, 100 rpm 60 s, 3000 rpm (Photoresist thickness: 1.7 µm)
Hotplate		85 °C, 2 min Rest for 10 min
Mask aligner	Mask type 2	10 s, 6 mW, hard contact mode
Hotplate		115 °C, 5 min Rest for 10 min
Mask aligner	Flood exposure	25 s, 6 mW
Reverse development	AR 300–26; DI	1:3, ca. 10 s
SRD	DI-water N_2_	700 rpm, 3 min 2000 rpm, 4 min
Microscope and FTP		Structure control

NOTE: Visual inspection for the photoresist structure with a microscope and Film Thickness Probe (FTP) is recommended.

Process parameters for hermetic passivation of BSK-glass for electronic device via PVD are shown in Table 11 and for passivation of BSK lift-off are shown in Table 12.

**Table 11:** Process parameters for hermetic passivation of BSK-glass for electronic device via PVD.

Process	Material	Parameters
Evaporation plant	BSK	250 Å, 2 Å/s, Power 24,5 %,

**Table 12:** Process parameters for passivation of BSK lifto-off.

Process	Material	Parameter
Acetone cleaning	Acetone Beaker 1 Acetone Beaker 2	5 min, 60 W 1 min, fresh Acetone
Cleaning bench	Isopropanol	Rinse off
QDR	DI	2 min
SRD	DI N_2_	700 rpm, 3 min 2000 rpm, 4 min
Plasma stripper	O_2_	40 % O_2_, 350 W, 5 min

## Gas phase silanization

After the completion of proper surface cleaning and activation, a resting period of 15 min was required to stabilize the active groups. Subsequently, the wafers underwent a silanization process in a desiccator situated inside a nitrogen-filled glovebox (GS GLOVEBOX Systemtechnik GmbH - Germany). Depending on the specific silane precursors used, (3-Aminopropyl)triethoxysilane (APTES) and 4-Aminothiophenol (ATP) (Sigma Aldrich), the concentration, volume, and temperature parameters were specified as shown in Table 13.

**Table 13:** Property parameter for (3-Aminopropyl)triethoxysilane (APTES) and 4-Aminothiophenol (ATP).

Silane	Purity [%]	ρ [g/mL]	Boiling point [ °C]	Salinization temperature [ °C]	Volume [µl]	Mass [mg]
(3-Aminopropyl) triethoxysilane	99	0.946	217	50	100	—
4-Aminothiphenol	9	1,17	no data	50	—	60

Inside the glovebox, the silane (ATP for Au, APTES/APS for SiO_2_/Si or glass) was placed into a crystal dish inside a pre-heated desiccator (50 °C). The wafer was oriented at an approximate angle of 85° relative to the outlet valve of the desiccator to maximize the contact area between the substrate and the silane-saturated gas stream. Then, the pressure inside the desiccator was reduced to 130 mbar, ensuring a continuous gas flow of the silane over the substrate. The process was carried out at 50 °C for 30 min and a silane linker layer was formed on the corresponding substrate surface. Subsequently, the silanized wafer was washed with ethanol (98 %) and DI water, and dried under a flow of N_2_ to remove any physically adsorbed impurities that may have been present during the silanization process. Then, the wafer was transferred to a second pre-heated desiccator to conduct a grafting procedure at 85 °C for 1 h to eliminate any remaining non-condensed leaving groups and thus ensure a complete reaction. Finally, the silane monolayer layer was formed on the substrate surface.

## Spin coating protocol for GO on wafer surfaces: an enabling platform for micro- and nano-structuring

Spin-coating for the creation of thin films was performed on either glass wafers or silicon substrates, with or without a 140 nm thick layer of dry oxide, following the suggested protocol. The same protocol was applied to the full surface Au-coated glass substrates, which were not micromechanically processed for the optical SPR. The cleaning, activation, and gas phase deposition methods described above were used for all sensor variants.

Approximately 6 mL of the GO dispersion (pH = 2.1) was pipetted onto the silane-layer-modified wafer until the surface was fully covered. The spin-coater was then immediately accelerated to a final speed of 3500 rpm without using a ramp. The diagram (Fig. 1, step III) illustrates the formation of hydrogen bonds between the functional carboxylic acid groups R_1—_COOH (R_1_ = nGO) and the amino groups R_2—_NH_2_ (R_2_ = C_3_H_6_SiO_3_/_2_) of the siloxanes. The configuration of the functional groups on individual GO flake base level and GO edge zones of the GO layers corresponds to a random distribution within the individual layer stack.

At the interface between the silane terminal groups and GO, amide bonds form between the silane amino groups and the carboxylic acid groups (R-COOH) of GO via condensation, leading to the formation of a closed layer on the wafer surface. A wafer baking step at 150 °C post-spin coating ensures complete condensation to form the amide bond across the entire wafer surface. All process steps and the changes in the surface's energy states were monitored via static contact angle and ellipsometry measurements to estimate the quality of the coating.

This bonding occurs across the entire 4″ surface, prompting all GO monolayers and GO flakes to react and form a cohesive thin film, which serves as the foundation for the creation of GO-based transducers. It's hypothesized that in addition to the amide bonds between the GO stack's basal planes and the silane layer, numerous weak non-covalent van der Waals interactions and hydrogen bonds are also at play, contributing to the stability of the transducers.

## Waferscale micro/nano patterning of GO thin-film

### Protocol for micro-structuring GO thin film (mask type 3) via reactive ion etching (RIE)

**Fig. 2:**
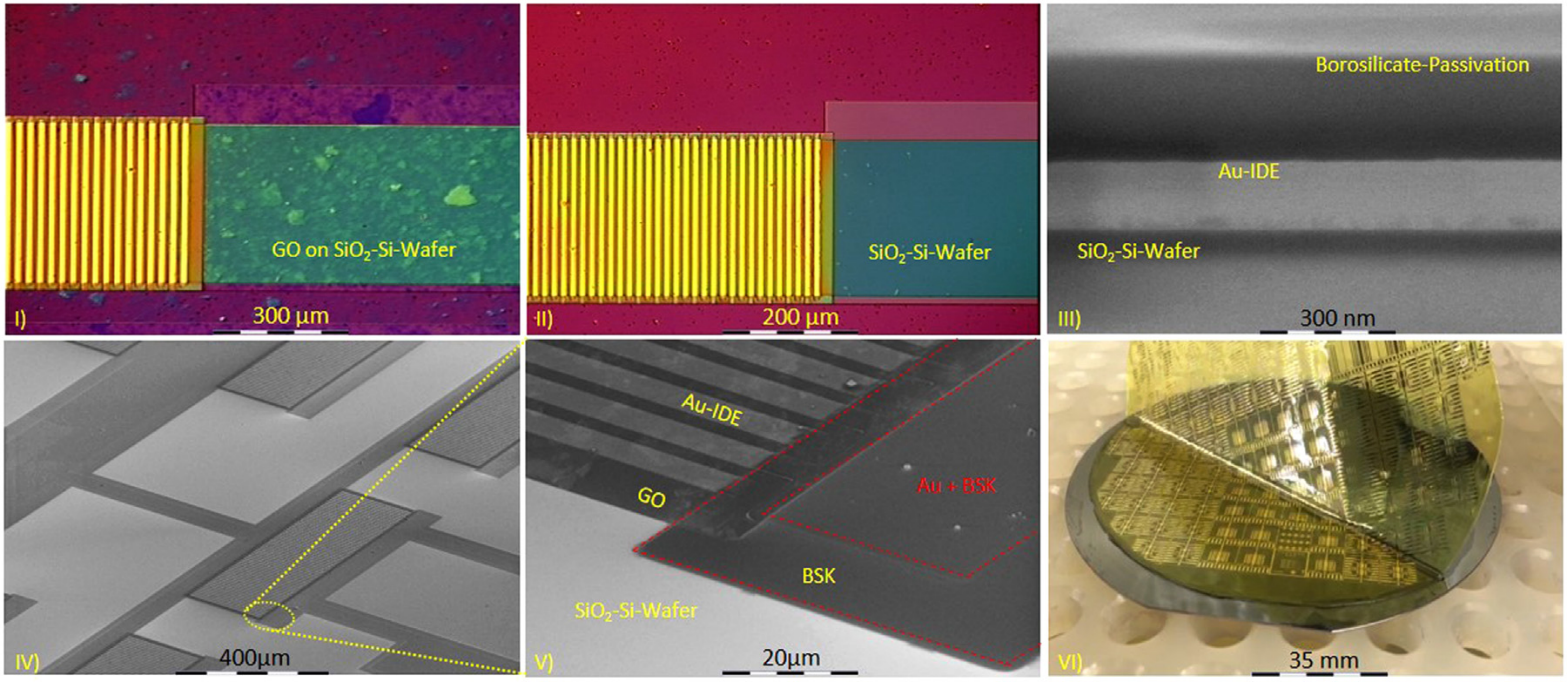
Waferscale micro/nano patterning of GO thin-film. I) shows before the structuring with the RIE process, the IDE junction, covered with photoresist as masking for the protection of the GO-based transducer (here to see the IDE: 256 fingers, channel length: 51,000 µm, GO transducer area: 200 µm x 2600 µm, IDE finger spacing: 5 µm, Au thickness: 140 nm, BSK thickness: 200 nm). The entire silicon area between the two conductor tracks, as well as the BSK passivation, is covered with GO-thin film. In II), the area etched by O_2_-RIE is revealed while preserving the IDE window masking. The cross-section III) shows the construction of the IDE electrode with hermetically sealed Au conductor tracks through deposited BSK passivation. Contrast display via SEM of the IDE structures (IV-V) (primary beam voltage 1.4 kV) illustrate how cleanly GO has been deposited and structured in the IDE. Image VI) can be seen as a prospect of how the manufacturing process described in the protocol, with identical parameters, including GO structuring, metallization, and BSK passivation, can also be transferred to 10 µm thick Polyimid foil substrate [8].

For electronic devices, GO thin films were patterned using standard lithography and O_2_ plasma reactive ion etching (RIE) processes (Sentech GmbH, Berlin, Germany) with mask type 3. The photoresist was then cleaned with acetone and isopropanol, each for 5 min. Referring to the following process guide, Fig. 2 highlights the results that impressively demonstrate the potential of the fabrication process for GO-based biosensors on both rigid and flexible substrates (Table 14).

**Table 14:** Process steps and parameters for wafer manufacturing (transferring the structures of mask type 3).

Process	Material	Parameter
Spin coater	Positive photoresist AR-U 4030	3 ml 7 s, 100 rpm 60 s, 3000 rpm (Thickness: 1.7 µm)
Hotplate		85 °C, 2 min Rest for 10 min
Mask aligner	Mask type 3	10 s, 6 mW, hard contact Mode
Reverse development	AR 300–26; DI	1:3, ca. 10 s
SRD	DI N_2_	700 rpm, 3 min 2000 rpm, 4 min
Microscope and FTP		Structure control
Reactive ion etching	O_2_-plasma	Reactor pressure: 15 Pa, 4,8 sccm O_2_, 100 W, 20 s
Microscope and FTP		Structure control

NOTE: Visual inspection for the photoresist structure with a microscope and Film Thickness Probe (FTP) is recommended.

### Nanoimprint lithography (NIL) protocol for GO thin film (mask type 4)

For the nanoimprint lithography process (Obducat Eitre6), a silicon master stamp was produced utilizing e-beam lithography. This stamp contains geometries with feature sizes that range from 300 nm to 100 nm, and a height of 80 nm. The stamp was then moulded into a spin-on PMMA polymer on silicon through the use of T-NIL. Optimal process parameters were thoroughly explored to ensure the integrity of the resulting structures.

**Fig. 3:**
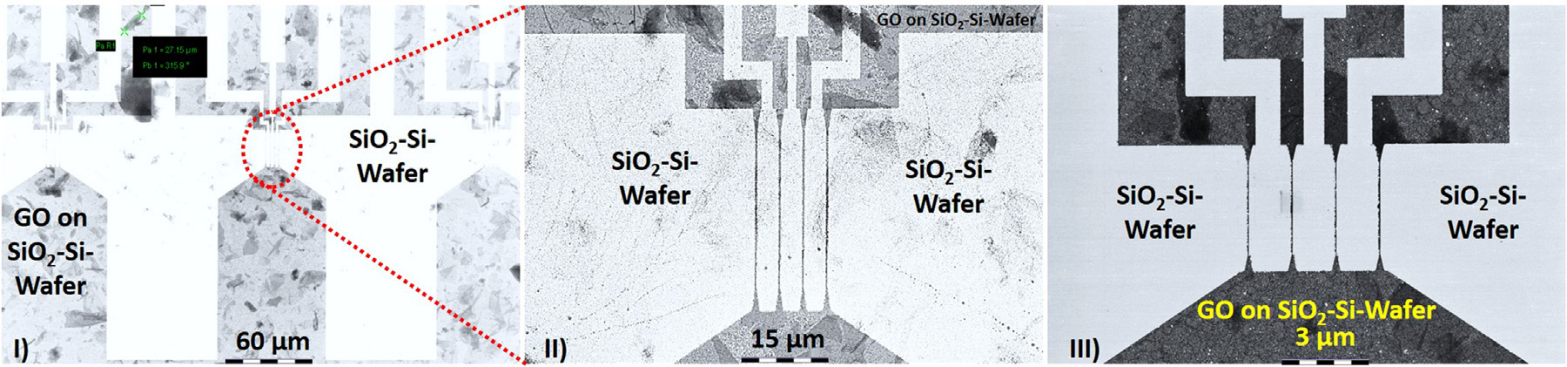
The SEM images (I)–(III) contrasts the GO nanoribbons at 3 keV, which could be fabricated using the detailed protocol from this publication. A silicon master stamp prepared for the nanoimprint process was used. With this NIL master, all GO nanoribbons were fabricated through Nanoimprint Lithography (NIL). The GO ribbons were etched via O2-RIE process. There are 4 GO nanoribbon electrodes between all source and drain accesses. The dimensions between source and drain in Figs. (I) and (II) have a length of 45 µm and a width of 150 nm. In III), the GO ribbon length is 4 µm with a width of 150 nm. The thickness of all GO nanoribbons ranges between 1 and 3 nm. It is interesting to note that in all representations, GO flakes have been incorporated into the thin film (darker regions), probably consisting of 1–3 layers of GO, which could not be completely separated during the LTED synthesis.

Following this, reactive ion etching with O_2_ was conducted to first remove the residual layer. Subsequently, the exposed GO layers were etched. The entire process provides a reliable and repeatable method for creating structured GO nanoribbons. The scanning electron microscopy (SEM) image contrasts the structures of the fabricated GO nanoribbons in Fig. 3 as a result of the described process (Table 15).

**Table 15:** Process steps and parameters for the nanoimprint lithography with a GO coated wafer.

Process	Material	Parameter
GO-wafer	Nanoimprint resist PMMA mr-I 7020R NIL 1000 Note: Use a particulate filter syringe 0,22µm	2–3 ml 30 s, 1000 rpm 60 s, 3000 rpm (Thickness: 200 nm)
Hotplate		100 °C, 1 min Rest for 10 min
FTP		200 nm ± 15nm
Nanoimprint	Silicon Master Stamp	Step 1: *p* = 40 bar, *T* = 115 °C *t* = 300 s Step 2: *T* = 115 °C, *p* = 1 bar, *t* = 5s
Microscope and FTP		Structure control 200 nm ± 15nm
Reactive ion etching	O_2_-plasma	Reactor pressure: 13.3 Pa, 3,1 sccm O_2_, 100 W, 35 s
Microscope		Structure control

NOTE: Visual inspection for the photoresist structure with a microscope and Film Thickness Probe (FTP) is recommended.

### Wafer dicing

Following the fabrication process, the 4-inch wafers were diced into suitable chip sizes. For SPR chips intended for use with the commercial Reichert SPR setup, the chips were cut to a size of 1.25 cm × 1.25 cm. For electrical sensors, the chips were cut to a size of 7 mm x 7 mm. To safeguard the chips during the dicing process, a layer of protective photoresist was spin-coated onto the wafers. Post dicing, the protective photoresist was removed by immersing the chips in acetone for 30 min at room temperature, then in fresh acetone, and finally in isopropanol for 5 min. The chips were dried with N_2_ as the final step.

### Thermal reduction and annealing

The thermal reduction of GO into rGO was executed at 350 °C for 10 h in a vacuum oven, followed by ambient annealing at 600 °C for 20 s. This subsequent annealing process was designed to facilitate the formation of ohmic contact between the microelectrodes and the rGO thin film, as well as to restore the density of oxygen functional groups on the surface of the rGO thin films, which is critical for biosensor applications. The parameters of this final annealing step, including the reduction temperature, duration, and gaseous environment, can be modulated as needed.

### Method validation

**Fig. 4:**
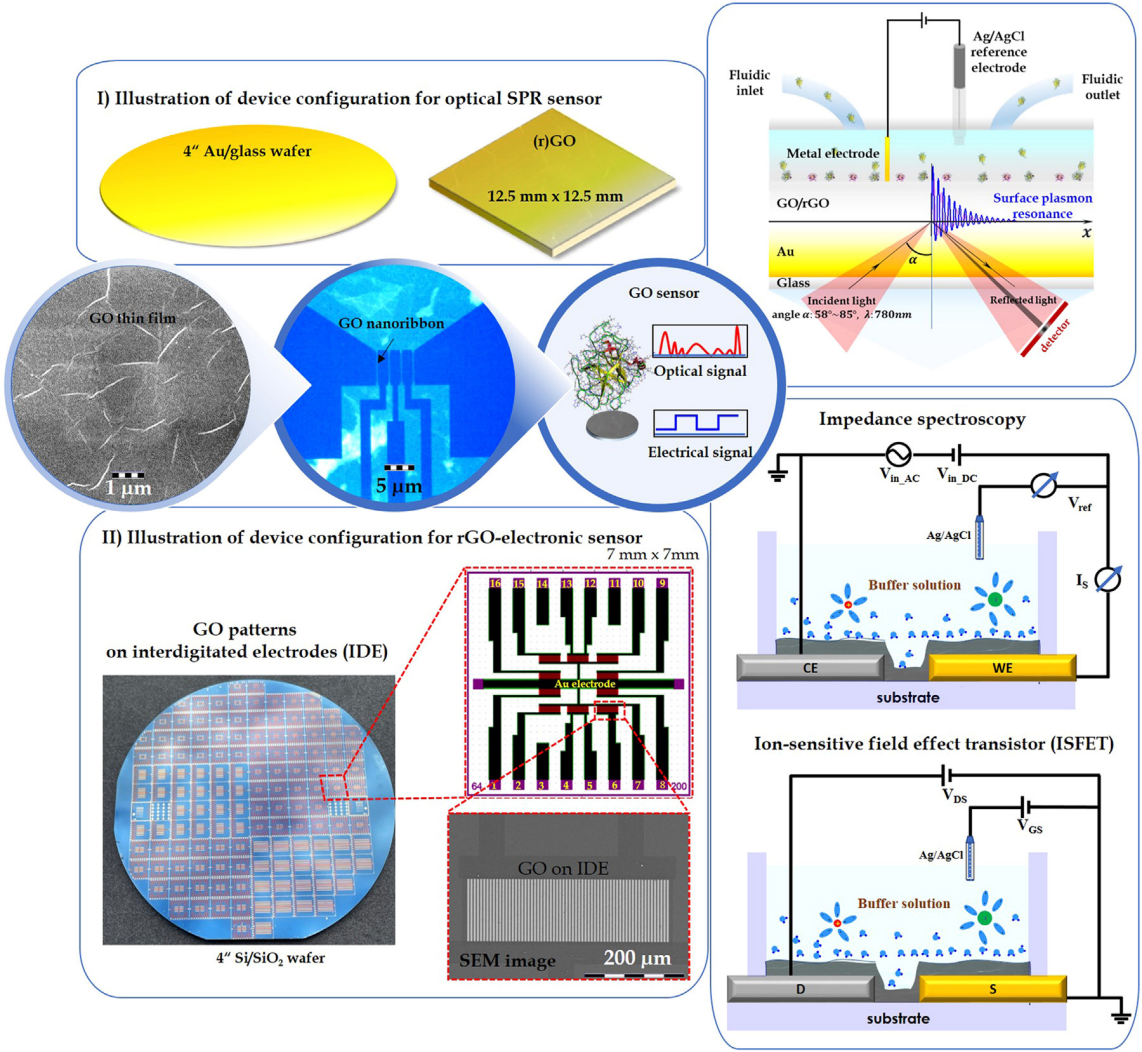
Illustrations of device configurations. I) (r)GO thin film was fabricated on a 4-inch Au/glass substrate for SPR biosensor applications. An electrochemical fluidic cell, which connects the SPR chip with the metal electrode and reference electrode, is optional. II) An rGO thin film or micro/nano pattern is integrated with microelectrodes on the 4-inch wafer for various sensor applications, such as ISFETs and impedance spectroscopy.

The process parameters for manufacturing GO-based thin film transducers have been outlined in our most recent process sequences for sensor applications, as seen in our publications [[1], [2], [3], [4],^6^] and are depicted in Fig. 4 (roadmaps I and II). These protocols have been continuously improved and updated over several years of research work. The protocol presented here offers a considerable level of reproducibility, with a yield of up to 96 % sensor functionality per wafer. Nonetheless, it's important to note that the production of GO and other graphene-based thin films on large wafer surfaces is still in the early stages of process standardization worldwide, and the manufacturing tolerances are yet to be as competitive as those seen in the semiconductor industry, such as with silicon-based, III-IV element-based transistors.

## Conclusions

The manuscript provides an in-depth insight into the fabrication of GO-based optical and electrical sensing systems with high sensitivity for large-area biomedical prototype development. The uniform and reproducible surface coating of wafers with GO remains crucial for all sensor configurations to be developed. With our technical methodology, a flat and continuous layer thickness between 1 and 3 nm can be achieved, and the subsequent structuring of micro- and nanopatterns through reactive ion beam etching with careful process control is significantly facilitated. This advance opens up numerous possibilities for sensor applications that require precise micro- and nanoscale processes and paves the way for the scalable production of GO-based sensors.

The protocol includes physical and/or chemical cleaning/activation methods, gas-phase silanization, micro/nanostructuring technologies, and thermal treatment. A major advantage is the manufacturing flexibility with high reproducibility of up to a 96 % device yield. It can be applied to various substrates such as solid surfaces made of gold, silicon or glass but also to flexible films such as polyimide and neopullium for various sensor applications. Using this presented manufacturing technology, the proof of concept studies of (r)GO sensors show exceptionally high sensitivity, large sensing dynamic ranges over several orders of magnitude, tunable SPR behavior and superior functionality in impedance spectroscopy and ISFETs device beyond the Debye screening limit. Multi types of biomarkers, protein, antigen, peptide, DNA and sugar such as lectin ConA, prostate-specific antigen (PSA), and NT-proBNP and glucose, were successfully detected in physiological buffer solutions (150 mM ionic strength) and human serum. These properties indicate significant potential for the commercialization of this GO-based transducers.

Moreover, combining 2D materials with other optical and electronic components requires tailored manufacturing strategies to ensure reproducibility and adherence to manufacturing tolerances, paving the way for product development in the near future. To improve the performance of the transducer, several considerations should be taken into account:

1) The development of a higher level of automation for GO spin coating-like batch processes required for large-scale industrial commercialization.
2) Using nanoscale lithographic processes to better compensate for variations in amorphous carbon (e.g., using a wafer stepper).
3) Optimization of the thermal reduction process to convert GO into rGO and restore the optical and electronic properties of rGO closer to those of pure graphene. This could be achieved by higher reduction temperatures in a vacuum or in a reducing gas environment.

The global production of GO and graphene-based thin films on large wafer surfaces is still at an early stage of process standardization. The manufacturing tolerances are not yet comparable to those in the semiconductor industry, for example, for silicon transistors manufactured in CMOS lines. Nevertheless, our protocol demonstrates the feasibility of scalability of graphene-based sensor fabrication, highlights the existing challenges that need to be addressed in further research, and lay the foundation for future research efforts and potential industrial applications in the biomedical field.

## CRediT authorship contribution statement

**Xiaoling Lu:** Methodology, Investigation, Writing – original draft, Writing – review & editing. **Walid-Madhat Munief:** Conceptualization, Methodology, Writing – original draft, Writing – review & editing. **Pavel Damborský:** Investigation. **Alice Kasjanow:** Investigation. **Jaroslav Katrlík:** Supervision, Writing – review & editing. **Vivek Pachauri:** Methodology, Supervision, Writing – review & editing. **Sven Ingebrandt:** Methodology, Supervision.

## Declaration of Competing Interest

The authors declare the following financial interests/personal relationships which may be considered as potential competing interests:

Walid-Madhat Munief reports financial support was provided by Euroimmun Medizinische Labordiagnostika AG, Germany. Vivek Pachauri reports financial support was provided by Euroimmun Medizinische Labordiagnostika AG, Germany.

## Data Availability

Data will be made available on request.
